# FACT: Functional annotation transfer between proteins with similar feature architectures

**DOI:** 10.1186/1471-2105-11-417

**Published:** 2010-08-09

**Authors:** Tina Koestler, Arndt von Haeseler, Ingo Ebersberger

**Affiliations:** 1 Center for Integrative Bioinformatics Vienna (CIBIV), Max F. Perutz Laboratories, Dr. Bohrgasse 9, A-1030 Vienna, Austria; 2 University of Vienna, Vienna, Austria; 3 Medical University of Vienna, Vienna, Austria; 4 University of Veterinary Medicine, Vienna, Austria

## Abstract

**Background:**

The increasing number of sequenced genomes provides the basis for exploring the genetic and functional diversity within the tree of life. Only a tiny fraction of the encoded proteins undergoes a thorough experimental characterization. For the remainder, bioinformatics annotation tools are the only means to infer their function. Exploiting significant sequence similarities to already characterized proteins, commonly taken as evidence for homology, is the prevalent method to deduce functional equivalence. Such methods fail when homologs are too diverged, or when they have assumed a different function. Finally, due to convergent evolution, functional equivalence is not necessarily linked to common ancestry. Therefore complementary approaches are required to identify functional equivalents.

**Results:**

We present the **F**eature **A**rchitecture **C**omparison **T**ool http://www.cibiv.at/FACT to search for functionally equivalent proteins. FACT uses the similarity between feature architectures of two proteins, i.e., the arrangements of functional domains, secondary structure elements and compositional properties, as a proxy for their functional equivalence. A scoring function measures feature architecture similarities, which enables searching for functional equivalents in entire proteomes. Our evaluation of 9,570 EC classified enzymes revealed that FACT, using the full feature, set outperformed the existing architecture-based approaches by identifying significantly more functional equivalents as highest scoring proteins. We show that FACT can identify functional equivalents that share no significant sequence similarity. However, when the highest scoring protein of FACT is also the protein with the highest local sequence similarity, it is in 99% of the cases functionally equivalent to the query. We demonstrate the versatility of FACT by identifying a missing link in the yeast glutathione metabolism and also by searching for the human GolgA5 equivalent in *Trypanosoma brucei*.

**Conclusions:**

FACT facilitates a quick and sensitive search for functionally equivalent proteins in entire proteomes. FACT is complementary to approaches using sequence similarity to identify proteins with the same function. Thus, FACT is particularly useful when functional equivalents need to be identified in evolutionarily distant species, or when functional equivalents are not homologous. The most reliable annotation transfers, however, are achieved when feature architecture similarity and sequence similarity are jointly taken into account.

## Background

The sequencing of entire genomes has become a routine task in molecular biology. To date, about 240 fully sequenced eukaryotic genomes comprising more than 3.7 Million protein coding sequences are available in the public domain [1]. Only a small fraction of these species are model organisms with considerably well characterized protein functions. Most of the remaining species are either of commercial or medical interest, qualify for new model organisms, or hold key positions required for the understanding of organismal evolution. The benefit of a newly sequenced organism essentially depends on the extent to which its data is integrated into existing knowledge about function and evolutionary relationships of genes in other species. A thorough experimental characterization of all proteins is not feasible. Therefore, comprehensive bioinformatics approaches are needed to reliably identify functionally equivalent proteins across species. Two roads are usually followed to accomplish this task.

The first and more common approach searches for proteins with a significant sequence similarity, which is commonly taken as evidence for their common ancestry. For example, a protein with unknown function can be used as query to search for similar sequences in annotated protein databases, e.g., with BLAST [2] or, for more sensitive searches, using machine learning algorithms, like PsiBLAST [2] or support vector machines [3-5]. The functional annotations of the best hits serve then as tentative annotations for the query, e.g., [6,7].

Clearly, one limitation is inherent in this approach: Functional equivalence is not tied to a significant sequence similarity. This can have several reasons: First, a query may not obtain a significant hit in a similarity search since the homologous proteins with the same function are too diverged, or are of low complexity. Second, homologs may be identified via sequence similarity but they have assumed diffrent functions [8,9]. For example, in the case of enzymes about 60% of sequence identity between homologous proteins is required to reliably infer functional equivalence [10,11]. Thus, a functional annotation transfer between homologs can be wrong. If such an error remains undetected, it can spread through databases [12]. Third, it has been shown that proteins with the same function are not always homologous, but rather are a result of convergent evolution [13]. In such instances sequence similarity based searches for functional equivalents produce no results. In summary, functional equivalence is not synonymous with homology. The second approach to identify functional equivalents does not rely on homology inference by means of pair-wise sequence similarity but rather considers other measures of protein similarity. Amino acid sequences can be annotated with a plethora of features, capturing different properties of the protein. Among others, these are functional domains, secondary structure elements and compositional properties.

The aggregate of all features in a protein constitutes its feature architecture, and it is supposed that this feature architecture allows conclusions about the function of a protein. A number of studies have shown the applicability of such a feature based approach, e.g., [14,15]. The possibility to deduce protein function from the feature architecture suggests that feature architecture similarity can be used to identify proteins sharing a similar function. For example, InParanoid displays the Pfam [16] domain annotation of homologous proteins [17]. Thus, we can quickly assess if homologs can be functional equivalents. In the same way, ProteinArchitect [18] finds similar proteins to a query sequence and displays the feature architecture of the hits. However, these tools provide the feature annotation only as an accessory information. The search for similar proteins in the first place is still performed on the amino acid sequence level. The necessity to include information about the feature architecture into the search for functional equivalents was emphasized by Forslund et al. 2008 [19]. They showed that roughly 12% of the feature architectures in 96 eukaryotic proteomes evolved more than once independently. Hence, the corresponding proteins are functionally similar although they are not homologous.

Despite its potential for identifying functionally equivalent proteins, only few strategies exploit the feature architecture for similarity searches [20-22]. Lin et al. [20] were the first to measure the similarity between feature architectures using a weighted sum of three indices. The first index measures the ratio of shared features to the total amount of features. The second index assesses the feature duplication similarity, and the third, the Goodman-Kruskal index, measures to what extent the same feature pairs occur in two proteins. A detailed description of the Lin score is given in the implementation section. Lee and Lee 2009 [22] additionally introduced a weighting scheme that reduces the influence of promiscuous Pfam domains [23]. Notably, all approaches share the same limitations. Most importantly, feature architectures are constructed only from Pfam domains. Thus, other features such as transmembrane regions or coiled coil domains indicative of protein function are ignored. Furthermore, the positional information of shared features in the compared proteins is not taken into account. Eventually, a systematic evaluation to what extent feature architecture similarity is helpful in detecting functional equivalents is also missing. Lin et al. 2006 [20] and Lee and Lee 2009 [22] evaluated their approaches only for their capability of detecting homologous proteins. Thus, the search for functional equivalents using feature architecture similarity is still in its infancy.

Here, we present FACT a comprehensive method for searching for functionally equivalent proteins using the criterion of feature architecture similarity. FACT considers a broad spectrum of features (functional domains, secondary structure elements, and compositional properties) to determine the feature architecture of a protein. Moreover, the positions of the features in a protein sequence are taken into account. FACT can be used to search for functional equivalents in entire proteomes and the credibility of the best hit is assessed by a p-value. This makes an automated large scale search for functional equivalents possible. A graphical interface, the feature dotplot, complements the automated similarity search and facilitates a visual comparison of two feature architectures. We evaluate the fidelity of FACT using a collection of EC classified enzymes and demonstrate FACT's applicability for identifying functional equivalents. A comparison to the performance of existing approaches to infer functional equivalence from feature architecture similarity, as e.g., described in [20,22], on the same set of enzymes is used to assess the improvement of FACT. Finally, we compare for the first time the usability of two protein similarity measures, sequence similarity and feature architecture similarity, for identifying functional equivalents, and we explore their respective strengths and weaknesses.

## Implementation

**Figure 1:**
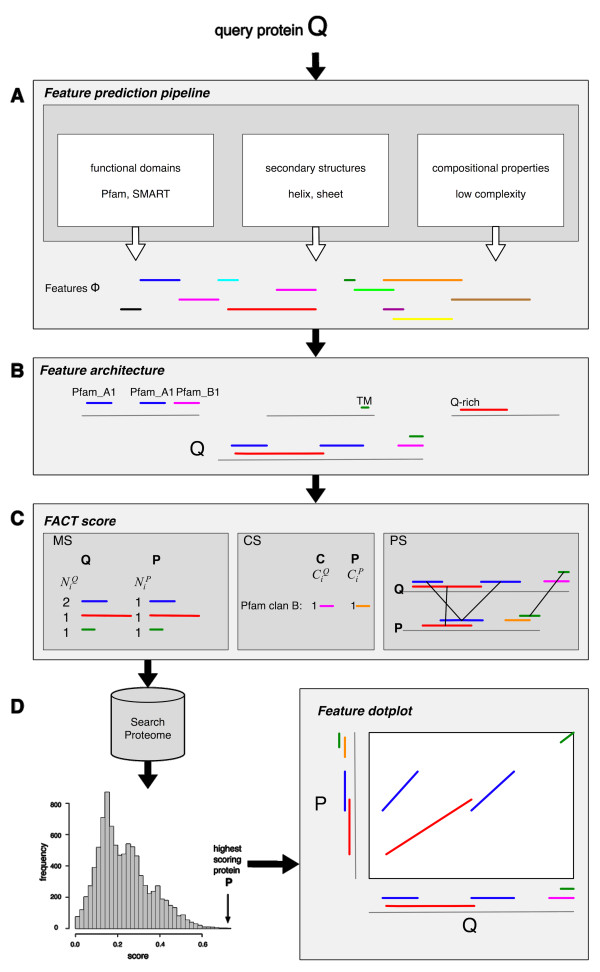
**Overview of FACT**. *(A) *The amino acid sequence of query protein *Q *serves as input for a collection of prediction programs, which annotate *Q *with features from Φ. *(B) *The assembly of instances from Φ constitute the feature architecture of a protein. *(C) *The *FACT *score captures the similarity between two feature architectures by a combination of the *Feature multiplicity similarity *(MS), the *Pfam clan similarity *(CS), and the *Feature positional similarity *(PS). The score is calculated between *Q *and every protein in a pre-annotated search proteome resulting in a list where the proteins in the search proteome are ranked in decreasing order according to their *FACT *score. *(D) *From the score list any protein *P *can be extracted and its feature architectures similarity to *Q *can be visualized in the feature dotplot.

As a first step, FACT annotates the query and each protein in the search set with a broad variety of features (Figure 1A), i.e., functional domains (Pfam domains, SMART domains [24], transmembrane regions, signal peptides), secondary structure elements (helix, strand, coiled coils), and compositional properties (low complexity regions, sequence composition). A pipeline of several feature prediction programs serves this purpose. The underlying feature set Φ is, therefore, determined by the collection of prediction programs. The feature architecture of a protein is then the arrangement of instances of features in Φ (Figure 1B).

### Measuring the similarity of feature architectures

To identify proteins with a similar feature architecture to a query protein *Q*, we measure the pairwise similarity between *Q *and every protein *P *in a proteome. We implemented a modified version of the score from Lin et al. 2006 [20] and introduce the *FACT *score.

#### Modified Lin score (MLS)

Lin et al. [20] score the similarity of two Pfam based feature architectures by combining the Jaccard index, a domain duplication similarity index, and the Goodman-Kruskal index with relative weights 0.36, 0.63, and 0.01, respectively. Calculating the Goodman-Kruskal index requires the order of Pfam domains along the sequence. Our feature set Φ contains a plethora of additional features that can overlap in the feature architecture (c.f. Figure 1B). In such instances, it is unclear how to assess the feature order. However, given its low relative weight of 0.01, the contribution of the Goodman-Kruskal index to the total score is negligible. Thus, we ignored this index in our implementation and adapted the weights of the two other indices accordingly. We calculate the MLS as

(1) $$\begin{array}{l} L(P,Q)=0.365*\frac{N^{PQ}}{N^{P}+N^{Q}-N^{PQ}}+\\ 0.635*\exp(-\sum_{i=1}^{N^{P}+N^{Q}}|N_{i}^{P}-N_{i}^{Q}|/N_{max}), \end{array}$$

where *N*^*PQ *^is the number of shared features between protein *P *and the query protein *Q. N*^*P *^and *N*^*Q *^are the number of different features in *P *and *Q*, respectively. $N_{i}^{P}$ and $N_{i}^{Q}$ count the instances of feature *i *in *P *and *Q*, respectively and

(2) $$N_{max}=\sum_{i=1}^{N^{P}+N^{Q}}max\left(N_{i}^{P},N_{i}^{Q}\right).$$

One drawback of the MLS is that it does not include information about the position of individual features in the proteins. Therefore, we introduce a new scoring function.

#### FACT score

The *FACT *score computes the feature architecture similarity between proteins as the weighted sum of three scores considering (i) the number of instances for all shared features, (ii) Pfam clan annotations, and (iii) the positions of shared features in the proteins. We describe the three building blocks of the *FACT *score in the following paragraphs.

##### Feature multiplicity similarity (MS)

The MS assesses to what extent the numbers of instances for a shared feature agree between two architectures. For each shared feature *i*, we compute the product of its number of instances in $P\;(\;N_{i}^{P}\;)$ and $Q\;(\;N_{i}^{Q}\;)$, and normalize this number by the theoretically maximal value $max{\left(N_{i}^{P},N_{i}^{Q}\right)}^{2}$. The MS is then the weighted sum over all shared features.

(3) $$MS(P,Q)=\sum_{i=1}^{N^{PQ}}\omega_{i}*\frac{N_{i}^{P}*N_{i}^{Q}}{max{\left(N_{i}^{P},N_{i}^{Q}\right)}^{2}},$$

where *ω*_*i *_> 0 is the weight for feature *i*. We use two weighting functions. First, *ω*_*i *_= 1/*N*^*Q *^where *i *= 1, ..., *N*^*PQ*^. This corresponds to an equal weighting of all features in *Q*. The resulting score is called MS_*uni*_. Second, we include the frequency of a feature *i *from *Q *in *P *into the weighting. To this end, $N_{i}^{P}$ counts how often feature *i *from *Q *is observed in *P*. The corresponding weight is then

(4) $$\omega_{i}=\frac{N_{i}^{P}}{\Sigma_{j=1}^{N^{Q}}N_{j}^{P}},$$

where *i *= (1, ..., *N*^*Q*^). This ensures that $\Sigma_{i=1}^{N^{Q}}\omega_{i}=1$. It is now straightforward to extend this weighting to a set of proteins {*P*_1_, *P*_2_, ..., *P*_*l*_}, e.g., a search proteome. We calculate the weight as

(5) $$\omega_{i}=\frac{\Sigma_{k=1\;\;}^{l}N_{i}^{P_{k}}}{\Sigma_{k=1\;\;}^{l}\Sigma_{j=1\;\;}^{NQ}N_{j}^{P_{k}}}.$$

We refer to this score as MS_*st*_. In the MS_*st*_, feature architectures sharing features that are rare in the search proteome receive a higher score than those sharing frequent features. This reflects the intuition that shared rare features are more likely to point towards a similar function than shared frequent features.

##### Pfam Clan Similarity (CS)

Pfam groups similar domains into clans [16]. For example, the Pfam clan RNase_H (CL0219) consists of 25 domains with a tertiary structure similar to that of Ribonuclease H. This structural similarity implies similarity in the function of the clan members. The CS score takes into account the co-occurrence of different Pfam domains in a clan. It is calculated analogously to the MS_*uni *_score.

(6) $$CS(P,Q)=\frac{1}{C^{Q}}\sum_{i=1}^{c^{PQ}}\frac{C_{i}^{P}*C_{i}^{Q}}{max{\left(C_{i}^{P},C_{i}^{Q}\right)}^{2}},$$

where *C*^*Q *^is the number of different Pfam clans in *Q*, *C*^*PQ *^is the number of shared Pfam clans between *P *and *Q*, and $C_{i}^{P}$ and $C_{i}^{Q}$are the numbers of instances of clan *i *in *P *and *Q*, respectively.

##### Feature positional similarity (PS)

The PS measures the distance between the relative positions a shared feature occupies in the compared proteins. For every instance of a shared feature in *P *and *Q*, we first determine the relative position within *P *and *Q*. Subsequently, we identify for each instance in *Q *the instance in protein *P *having minimal distance. One minus the minimal distance between two feature instances yields a similarity. We calculate PS as following

(7) $$PS(P,Q)=\sum_{i=1}^{N^{PQ}}\frac{\omega_{i}}{N_{i}^{Q}}\sum_{j=1}^{N_{i}^{Q}}\left(1-\underset{1\leq l\leq N_{i}^{P}}{\min}|q_{j}-p_{l}|\right),$$

where the relative position *q*_*j *_of the *j*^*th *^instance of feature *i *in protein *Q *is the center position of this instance divided by the sequence length. The positions *p*_*l *_of the feature instances in protein *P *are calculated accordingly. The use of relative positions ensures that shared features located at the C-terminus in both proteins have a small distance even if the protein lengths are different. The weights *ω*_*i *_of the individual features correspond to those of the MS_*st*_.

The *FACT *score is a weighted linear combination of the *Feature multiplicity similarity *(MS_*st*_), the *Pfam clan similarity *(CS), and the *Feature position similarity *(PS) (Figure 1C).

(8) $$FACT=\alpha *MS_{st}+\beta *CS+\gamma *PS\in \left[0,1\right],$$

where *α *+ *β *+ *γ *= 1, and *α*, *β*, *γ *≥ 0.

### Score statistics

**Figure 2:**
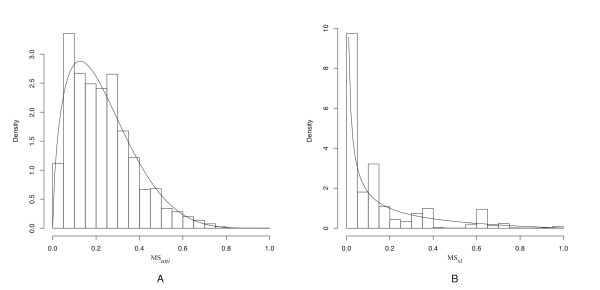
**Fit of the beta distribution to the score histograms**. Shown are typical score histograms from two FACT searches in the *T. brucei *proteome using the scoring function *(A) *MS_*uni*_, and (B) MS_*st*_. Despite the different shapes of the histograms, the beta distribution displays in both cases a good fit to the data.

Using the scoring functions introduced in the previous section, we calculate the feature architecture similarity scores between a query protein and every protein in a search proteome. From the resulting distribution of scores, we assess the extent to which the top scoring protein stands out from the lower ranking proteins (Figure 1D). For this purpose, we fit a beta distribution [25] to the score histogram. We have chosen the beta distribution for two reasons. First, it can assume different shapes. This fits well with histograms, even when different scoring functions are used (Figure 2). Second, it is defined in the range from 0 to 1, which is the exact range of the scores. We estimate the two shape parameters of the beta distribution from the mean, and the variance from all scores by the method-of-moments [25]. The p-value for a score *x *is then calculated as one minus the cumulative distribution function of the beta distribution of *x*. The smaller the p-value is, the more pronounced is the feature architecture similarity between the query and the highest scoring protein compared to that of the lower ranking proteins.

### Feature Dotplot

For a visual inspection of individual query hit pairs, we have developed the *feature dotplot *(FDP, Figure 1D), which extends the idea of a classical dotplot to the feature level. The FDP projects the features of two proteins along the x-and y-axis, respectively. A feature occurring in both proteins is represented by a diagonal line in the dotplot, where the slope of the line indicates the length ratio of the features in the proteins. Different features are represented by different colors. The standard amino acid dotplot is embedded into the FDP as well.

### FACT webpage

FACT is provided online on the webpage http://www.cibiv.at/FACT. The user can search for functional equivalents to a query protein in entire proteomes. Currently, the collection consists of 26 eukaryotic species (13 animals, 7 fungi, 3 plants, and 3 protists), but further species will be added. For every query protein FACT determines the feature architecture. Then the *FACT*, MS_*uni*_, MS_*st *_and the MLS scores between the query protein and all proteins in a search proteome are computed. For each scoring function the 100 highest scoring proteins are listed and a histogram of all scores is displayed. Additionally, the p-values for the highest scoring protein are shown. The FDP between the query protein and any protein from the score list can be viewed. The FDP links Pfam and SMART domains to the corresponding web pages. Furthermore, possibilities for displaying or hiding specific features, changing the word size for the amino acid dotplot and for exporting the feature dotplot are provided. Finally, a BLAST search against the search proteome is performed and the best three hits are listed. A FACT search conducted on this webpage takes currently about 1 to 5 minutes depending on the size of the search proteome. As an alternative to the proteome wide search, the FDP can be used to compare two user-defined proteins. The features of both sequences are annotated automatically and displayed in the dotplot.

## Results

FACT has been developed for identifying functionally equivalent proteins. To assess the applicability of our program we require that the tested proteins have the exact function assigned. To the best of our knowledge, only the proteins annotated by the Enzyme Commission (EC) satisfy this condition. The EC provides a hierarchical classification of the reaction catalyzed by an enzyme. The code consists of four numbers separated by dots. The first number determines the main catalyzed reaction (1 = Oxidoreductases, 2 = Transferase, 3 = Hydrolases, 4 = Lyases, 5 = Isomerases, 6 = Ligases), while the last number provides the most specific information about the catalyzed reaction. If two enzymes share the same EC number, they catalyze the same reaction and are therefore functional equivalents. Thus, the EC classifies enzymes according to their function and not according to their evolutionary relationships (c.f., [13]). We collected EC annotated proteins from human, fly, worm, yeast, and arabidopsis and filtered the dataset such that each EC number is represented at least twice. The final test set is comprised of 9,570 proteins. The average and median numbers of proteins with the same EC number are 10 and 4, respectively.

### Comparison of different scoring functions

For our evaluation, each protein from the test set served as a query for FACT. The similarity scores between the query protein and the remaining 9,569 proteins from the test set were then calculated. Subsequently, we compared the EC number of the highest scoring protein(s) to that of the query. If one highest scoring protein has the same EC number as the query, the proteins are functional equivalents. The fidelity of a scoring function is then the percentage of searches where a functional equivalent gets the best score. Table 1 shows the fidelities for the different scoring functions. For the *FACT *score we chose *α*, *β *and *γ *in the ratio 3:1:1 (cf. equation 8). The MLS and MS_*uni *_display fidelities of around 80%, thus in 20% of the 9,570 searches a protein that is not functionally equivalent to the query obtains the highest score. Weighting the individual features according to their frequency in the test set (MS_*st*_) increases the fidelity to 83%. The best result was obtained with the *FACT *score which also takes clan similarity and positional information into account. In 8,017 out of 9,570 cases (84%), a functional equivalent to the query obtained the highest score.

**Table 1:** Fidelity of FACT using different scoring functions.

scoring function	# prot (%)
MLS (Eq. 1)	7,685 (80.30)
MS_*uni *_(Eq. 3)	7,712 (80.59)
MS_*st *_(Eq. 3, 5)	7,908 (82.63)
*FACT *(Eq. 8)	8,017 (83.77)

'# prot (%)' denotes the number and percentage of correctly identified functional equivalents in the test set of 9,570 proteins.

When we analyzed the fidelity with respect to the main reaction catalyzed (first digit of the EC number), a functional equivalent was identified for 9,018 query proteins (94%; *FACT *score).

### Relevance of features

In addition to Pfam and SMART domains, the underlying feature set Φ of FACT includes a variety of other protein features, e.g., secondary structure elements and compositional properties. We next assessed the relevance of including these features. We compared the fidelity of the functional equivalent search using Pfam domains only to the fidelity based on the full feature set. The median number of proteins having the same best score is 9-13 (depending on the scoring function) for the Pfam only set. This number decreases to 1 for the full feature set. Thus, considering a broad variety of features leads to a better discrimination in the assessment of feature architecture similarity. In contrast, searches using only Pfam domains frequently end up with many equally best scoring proteins representing different EC numbers. For further evaluation, we consider a hit protein only then as an identified functional equivalent when its EC number matches that of the query, and additionally when it is uniquely top ranked in the score list. Table 2 shows the results of this analysis. The fidelities for the Pfam only set range, depending on the scoring function, between 6 and 9%. Using the full feature set leads to a drastic increase of the fidelity to values between 58 and 74%.

**Table 2:** Fidelity of FACT depending on the feature set.

	Pfam domains	all features
scoring function	# prot (%)	# prot (%)
MLS	891 (9.31)	5,618 (59.70)
MS_*uni*_	572 (5.98)	5,592 (58.43)
MS_*st*_	594 (6.21)	5,792 (60.52)
*FACT*	-	7,091 (74.10)

'# prot (%)' denotes the number of top ranked functional equivalents with a unique highest score. Since the *FACT *scoring considers clan information it was not used for the calculation with only Pfam domains.

### p-value for FACT hits

For each highest scoring protein a p-value is calculated. We determined the relation between p-value and the fidelity of FACT using the *FACT *score. With a decreasing p-value, the fidelity increases to a maximum of 98% at a p-value smaller than 10^-11 ^(see Additional file 1, Figure S1). Considering only those functional equivalents as identified that are uniquely top ranked, the fidelity increases up to 85% at a p-value below 10^-9 ^. However, as expected, the increased fidelity comes at the cost of the coverage. For example, of the 9,570 searches only 1,558 have a highest scoring protein with a p-value smaller than 10^-9 ^(see Additional file 1, Table S1). Our analysis shows that an annotation transfer between the query hit pair becomes more reliable when the p-value is small. Thus, we conclude that the choice of the beta distribution leads to sensible results.

### Feature architecture similarity vs. sequence similarity as a proxy for functional equivalence

**Figure 3:**
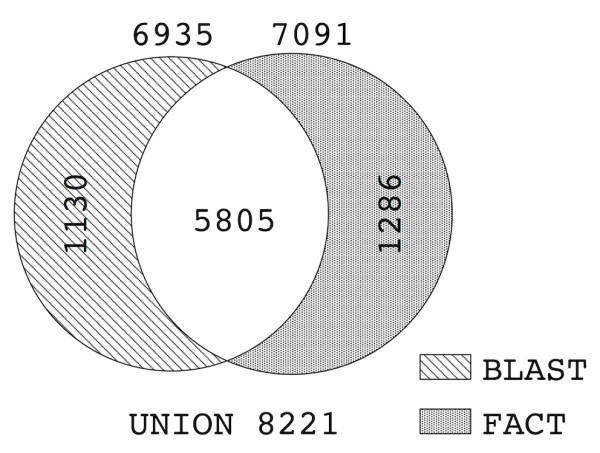
**Venn diagram contrasting the performance of FACT (FACT score) and BLAST on the test set**. Given are the the numbers of uniquely top ranking proteins having the same EC number as the query. For 1,286 (FACT) and 1,130 (BLAST) queries, respectively, only one program identified a functional equivalent.

With FACT we provide a comprehensive tool to search for functional equivalents using feature architecture similarity. We now compare FACT to the alternative approach that identifies functional equivalents via a significant sequence similarity, e.g., using BLAST [6,7]. Therefore, we run both methods on the test set. To ease the comparison between the two results, we again required a correctly identified functional equivalent to be uniquely top ranked. Figure 3 breaks down the results from BLAST and FACT (*FACT *score). BLAST identified 6,935 (72.5%) functional equivalents compared to 7,091 (74.1%) for FACT. In 5,805 (60.7%) searches both approaches obtained a functional equivalent as highest scoring protein. Moreover, in 4,017 (42%) searches the highest scoring proteins were even identical. 1,286 (13.4%) functional equivalents were detected exclusively by FACT, whereas 1,130 (11.8%) were detected only by BLAST. Although FACT performs slightly better than BLAST, the large number of functional equivalents found only by BLAST indicates that both approaches are complementary. This conjecture is further corroborated by the following observation: When FACT and BLAST detect the same protein as best hit, the query hit pair is in 99% of the cases functionally equivalent.

FACT outperforms BLAST in situations where sequence similarity between functional equivalents is low. When the E-value exceeds one, the best BLAST hit is only in 1% a functional equivalent. For the same query proteins FACT still achieves a fidelity of 31% (Additional file 1, Figure S2). For E-values ≥10^-20 ^the fidelity of BLAST increases to 39%, but it is still higher for FACT (46%).

**Figure 4:**
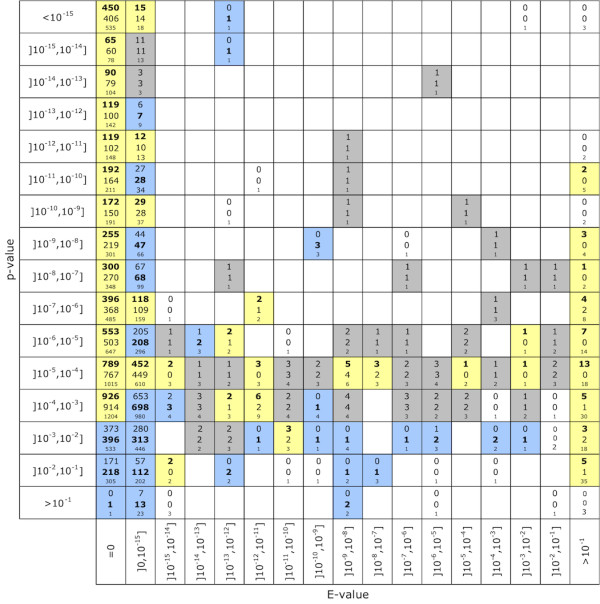
**Contrast of BLAST and FACT (FACT score) for different E-value/p-value combinations**. The matrix bins the 9,570 proteins according to the E-value and the p-value of the best hit when used as query for BLAST and FACT, respectively. The total number of proteins for a E-value/p-value combination is given by the bottom number in the corresponding cell. The two further numbers in a cell give the number of searches FACT (top) and BLAST (middle) had a functional equivalent as top scoring protein. The count number for the better performing tool is given in bold face. Yellow cells show E-value/p-value combinations where FACT identified more functional equivalents than BLAST, whereas the blue cells indicate a higher fidelity of BLAST. Grey cells mark ties.

To further explore the complementarity of both approaches we conducted a more detailed analysis. For any query protein in our test set, BLAST and FACT each identified a top scoring protein with an associated E-value and p-value, respectively. First we showed that E-value and p-value are not correlated (Pearson correlation cofficient: 0.09). Thus, a query obtaining a BLAST hit with a small E-value does not imply a FACT hit with a small p-value, and vice versa. Second, we binned the query proteins according to their E-value/p-value combination. For each combination, we counted the number of query proteins that fall into the bin. Then for each bin we counted how often BLAST and FACT identified a functional equivalent. These numbers are represented in the matrix shown in Figure 4. This matrix gives a guideline under which E-value/p-value combination either BLAST or FACT is more likely to find a functional equivalent. For query proteins obtaining poor BLAST hits (E-value > 0.1), the FACT predictions are more credible. A similar picture emerges for queries having a BLAST hit with a reported E-value of zero. Once the p-value exceeds 10^-3^, FACT always identifies more functional equivalents than BLAST. Finally, we note that PsiBLAST is more sensitive than BLAST in detecting even weak sequence similarities that may be indicative of a similar function. We therefore compared FACT also to PsiBLAST. This confirmed our findings from the FACT-BLAST comparison (see Additional file 1, Figures S3 and S4).

### Example applications of FACT

To illustrate the versatility of FACT in searching for functional equivalents we discuss two examples. The general procedure of a FACT search is summarized in Figure 1.

#### Missing link in the glutathione metabolic pathway

**Figure 5:**
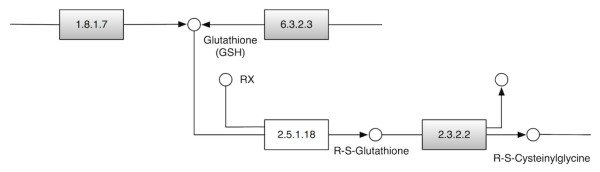
**Section of the KEGG glutathione metabolic pathway (ko00480)**. Grey filled boxes represent proteins of the human pathway for which KEGG orthologs exist in *S. cerevisiae*. An ortholog to the human glutathione S-transferase (EC 2.5.1.18), a central component of this pathway, could not be identified in yeast.

A common task in comparative genomics is the identification of proteins that are involved in known metabolic pathways in different species. As of today, the evolutionary relationships between proteins are usually used for this purpose, e.g., [26]. In some cases however, orthologs to individual proteins cannot be identified. Consequently, the question is raised of whether the corresponding functional equivalents are not present in the respective species or whether sequence similarity based searches cannot detect them. The glutathione metabolic pathway in the KEGG database [26] constitutes one illustrative example. It is one of the central detoxification pathways in animals and fungi. An ortholog to the human glutathione S-transferase (EC number 2.5.1.18), a central enzyme in this pathway, is not annotated in the yeast genome. However, orthologs to the human proteins flanking the glutathione S-transferase in the pathway are present (Figure 5).

**Figure 6:**
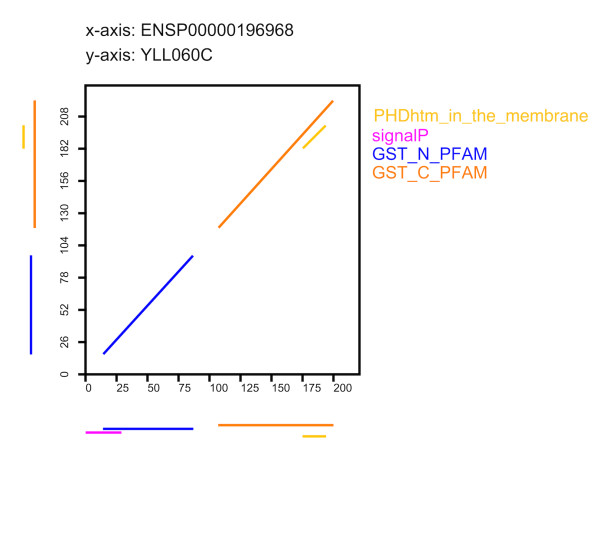
**Feature dotplot of the human glutathione S-transferase and the best FACT hit in yeast**. The two proteins share the Pfam domains GST_N (PF02798) and GST_C (PF00043), as well as a transmembrane domain. A signal peptide (signalP) is present only in the human protein. For better readability *α *helix and *β *sheet annotations are not shown.

A BLAST search using the human glutathione S-transferase protein as query revealed no significant hits in the yeast proteome. The best BLAST hit (YNL286W; E-value = 0.51) has no feature in common with the human query protein except *α*-helices and *β*-sheets. Instead, it contains two RNA recognition motifs (RRM_1). Similarly, the best PsiBLAST hit (YCL009C; E-value = 1.3) has no feature in common with the human query protein except *α*-helices and *β*-sheets. Next, we performed a FACT search in the yeast proteome, again with the human enzyme as query. This revealed the same best hit for all scoring functions (YLL060C; *FACT *score: p-value = 3 × 10^-6^). Thus, from the corresponding E-value/p-value entry in Figure 4 (>10^-1 ^/]10^-6 ^, 10^-5^]), there is a 50% chance of having detected a functional equivalent. We next used the FDP of the FACT hit and the query protein to validate the candidate (Figure 6). Both proteins have the N-terminal and the C-terminal glutathione S-transferase (GST) domains and share a predicted transmembrane region. Therefore, we conclude that the two proteins are functional equivalents. This has indeed been confirmed, since both proteins have been annotated with the same EC number [27]. Thus, FACT helps to identify candidate proteins that may close gaps in biochemical pathways.

#### Functional equivalents for GolgA5

In our second example we focussed on a structural protein, GolgA5, which is important for assembling and maintaining the structure of the human golgi apparatus [28,29]. Almost the entire protein is made up of coiled coils. This structure is formed by low complexity repeat units consisting of hydrophobic and polar residues. Consequently, many different sequences can assume a coiled coil structure. Thus, a sequence similarity based search for functional equivalents in very distantly related species is likely to be not successful. We performed a FACT search with the human GolgA5 in *Trypanosoma brucei*. The highest scoring protein agrees only between the MS_*st *_and the FACT score (Tb11.02.5040), while two different proteins were identified by the MS _*uni *_(Tb11.02.4670) and the MLS (Tb927.5.1900). For that reason, all top ranked hits and the best BLAST hit (Tb11.52.0008) and best PsiBLAST hit (Tb927.7.3330) were analyzed with the FDP (see Additional file 1, Figure S5-S8. The FDP of the PsiBLAST hit is not shown since this protein is 4,334 aa in length.) Since the function of GolgA5 requires its anchoring in the plasma membrane, we curated the results according to the presence of a transmembrane region. Among all hits, Tb11.02.4670 (MS_*uni*_) is the only protein that shares a C-terminal transmembrane region with the human GolgA5. Thus, we consider it to be the most promising candidate for the GolgA5 functional equivalent in *T. brucei*. Notably, it was recently shown that this protein exerts the same function in *T. brucei *as GolgA5 does in humans [30].

## Discussion

Here we present FACT, a tool for searching for functionally equivalent proteins. FACT computes the pairwise similarity between feature architectures and identifies for a query protein the highest scoring hit in an entire proteome. Evaluating the performance of FACT on EC classified enzymes reveals a fidelity of 84%.

How to measure the similarity between feature architectures still remains an open question. So far, all suggestions are ad-hoc solutions to the scoring problem. For example, the Lin score [20] assesses the similarity between two proteins from their features in common and also considers the set difference. Thus, features that are not shared between two proteins reduce the score. This scoring appears reasonable when feature architectures consist only of functional domains, e.g., Pfam domains. In such cases, the presence of an extra feature in one protein is likely to also reflect a functional difference between the compared proteins. However, in our study we used a comprehensive feature set, where some features lack an obvious connection to function. Therefore, we introduce a new score that considers only shared features. Our evaluation on a set of EC classified enzymes reveals that the fidelity in identifying functional equivalents does not heavily depend on whether or not the feature set difference between two proteins is taken into account. Both scoring functions, the MLS and MS_*uni *_perform equally well. Their conceptual difference, however, becomes relevant in individual cases as shown by our GolgA5 example application. The best scoring protein according to the MLS shares 4 features with the query and has one extra feature (c.f. Additional file 1, Figure S3). In contrast, MS_*uni *_identifies a highest scoring protein that shares 5 features with the query but has 4 extra features (c.f. Additional file 1, Figure S4).

The idea of giving individual features different weights has been presented before. Lee and Lee 2009 [22] weight a Pfam domain depending on its frequency in the RefSeq database [31] and on the diversity of its flanking Pfam domains. Note that the latter criterion is not straightforward to implement when features can fully overlap, and hence, feature order cannot be determined. In the MS_*st*_, we weight a feature according to its inverse frequency in the search proteome. This weighting scheme can be applied to any feature, and takes into account that feature frequencies can vary between search proteomes. The comparison of MS_*uni *_and MS_*st *_reveals that the introduction of weighting increases the fidelity by 2%. Unfortunately, comparing the effect of our weighting to that of Lee and Lee 2009 [22] is impossible, since in their evaluation the scoring functions differed not only in the weighting but also in the way shared domains are scored.

Among all scoring functions, the *FACT *score performs best (Table 1 and 2). This is the consequence of including clan similarity and positional similarity. We compute the *FACT *score by combining the scoring functions MS_*st*_, CS, and PS in a ratio of 3:1:1. We consider the number of shared features and their number of instances to be the most important parameters in determining the similarity between feature architectures. The clan annotation as well as the position of features are supplementary information that only have a moderate influence on the final score. Note that we deliberately did not optimize the weight parameters *α*, *β*, and *γ *with respect to the fidelity on the EC based functional annotation. Enzymes cover only a fraction of the diversity of protein functions. We wanted to avoid a bias towards this particular class of proteins, which could interfere with the general applicability of FACT [32].

In contrast to existing tools that use Pfam domains for identifying functionally similar proteins [20,22], FACT recruits a diverse set of features for building the feature architectures. Our evaluation highlights the significance of using a comprehensive feature set. When considering only Pfam domains, the median number of equally best scoring proteins is 9-13, depending on the scoring function. The most extreme case comprises the 589 enzymes lacking any Pfam domain. When these proteins are used as query, all proteins in the search proteome obtain the same score. However, the median number of enzymes with the same EC number as the query is only 3. Consequently, in the vast majority of searches more than one EC number is represented by the top ranked proteins. The search result is therefore ambiguous. To facilitate a meaningful assessment of the fidelity, we required a correctly identified functional equivalent to be uniquely top ranked. This reveals a maximal fidelity of 9% (Table 2). In contrast, when we use the FACT feature set, the median number of equally best scoring proteins reduces to one. This shows that the similarity score becomes more discriminative when more features are considered. As a consequence, the fidelity raises to 74% (*FACT *score). Notably, for the proteins without Pfam domains a correct functional equivalent was still identified in 158 cases.

There is still room for improvement regarding the search for functional equivalents. So far, all approaches are based on ad-hoc solutions for measuring the similarity between feature architectures since modeling their evolution is still an open problem. Moreover, the function of a protein essentially depends on its tertiary structure. However, tertiary structure elements are not yet part of the feature set. Both the integration of evolutionary models and of complex features is likely to result in more sensible similarity measures.

Feature architecture similarity based approaches identify functional equivalents. This supposedly complements sequence similarity based approaches represented, e.g., by BLAST or PsiBLAST. Here we have compared the fidelity of FACT to that of BLAST. A substantial fraction of functional equivalents were top ranked exclusively by FACT. This includes the cases where sequence similarity was too low to result in a significant BLAST hit, but FACT still detected functional equivalents. Finally, we observed no linear correlation between the E-value of the best BLAST hit and the p-value of the best FACT hit for a given query. In summary, these results confirm the complementarity of feature architecture similarity based approaches and sequence similarity based approaches in the search for functional equivalents. This finding is independent of whether we used BLAST or PsiBLAST. The complementarity is further corroborated by those searches where FACT and BLAST identify the same best hit. In such instances, the fidelity increases to 99%. Thus, a joint application of a feature architecture measure and a sequence similarity measure allows for highly reliable automated functional annotation transfers. However, this increase of the fidelity comes at the cost of detecting only 42% of the present functional equivalents in our test data. In cases where the two approaches disagree, we need to decide which of the hits is more likely to be a functional equivalent. To facilitate this decision, we have compared the fidelities of BLAST/PsiBLAST and FACT depending on the E-value and p-value of the highest scoring protein for a given query (c.f. Figure 4, Additional file 1, Figure S4). Notably, for searches where both methods obtained a good hit, i.e., small E-value and small p-value, respectively, FACT finds a functional equivalent more often than the other program. However, in most cases, a decision of whether a FACT hit that is not confirmed by BLAST, or vice versa, is a functional equivalent will require manual curation. We have presented two examples where we searched for functional equivalents to the human glutathion S-transferase in yeast, and to the human GolgA5 in *T. brucei*. These examples showed that the feature dotplot is a versatile tool to curate results from FACT searches. The feature dotplot facilitates an educated judgement of how similar two feature architectures are, and how likely it is that the corresponding proteins are functionally equivalent. Together with the implementation of four different scoring functions and the BLAST search, the feature dotplot complements the toolbox for a comprehensive search for functional equivalents.

## Conclusions

FACT uses the similarity of feature architectures between two proteins to search for functional equivalents in entire proteomes. FACT has a high fidelity and outperforms existing approaches that identify functional equivalents based on the presence of PFAM domains. This increase in fidelity is mainly accomplished by using a diverse set of features that are recruited for building the feature architectures. The different weighting of individual features and the relative position of shared features in the compared proteins provide additional information. FACT complements sequence similarity based approaches, such as BLAST, in the search for proteins with an equivalent function. It is, thus, particularly useful when distantly related species with highly diverged sequences are analyzed, or in cases where functional equivalents are not homologous. Both aspects will become increasingly relevant the more genome data from 'exotic' species becomes available. However, there exists no globally optimal solution to the problem of identifying functionally equivalent proteins. It is therefore necessary to compare the results from different scoring functions measuring feature architecture similarity and from sequence similarity based searches to select the most promising functional equivalent candidates. The feature dotplot to visually inspect the feature architectures of two proteins facilitates this manual curation. We have demonstrated the joint use of FACT, BLAST and the feature dotplot in a comprehensive search for functional equivalents in two example applications. They serve as a guideline of how to use these tools to propagate existing knowledge about the function of proteins from one species to another.

## Methods

### FACT

FACT annotates functional domains, secondary structure elements and compositional properties in protein sequences using the tools in the Sfinx package [33]. Low complexity regions are identified with the program seq, helices and strands with the program *PHDseq*, coiled coils with the program *COILS2*, and signal peptides with the program *SignalP*. Transmembrane regions are predicted both with *TMHMM *and *PHDhtm*. Pfam (version 23; [34]) and SMART (smart_16_04_2008; [24]) domains are identified with HMMER2 http://hmmer.janelia.org/ and regions enriched for a particular amino acid with CAST [35]. Pfam clan information was downloaded from http://pfam.sanger.ac.uk/. All annotation results are transformed into the SFS format [33]. This data structure allows for an easy extension of the feature set with features currently not considered by FACT. For sequence similarity searches BLAST version 2.2.13 and PsiBLAST version 2.2.23 was used. PsiBLAST searches were run with default parameter settings using 5 iterations. The FDP is implemented as a Java applet requiring Java 1.5 or higher. It can be accessed with a web browser with Java and with Java script enabled.

### Test set

We compiled the test set for the FACT evaluation using an initial collection of 9897 EC annotated enzymes from *Homo sapiens *(6,339), *Arabidopsis thaliana *(1,156), *Saccharomyces cerevisiae *(1,099), *Drosophila melanogaster *(896) and *Caenorhabditis elegans *(407). Protein sequences were downloaded from Ensembl 52 (*D. melanogaster*, *C. elegans*, *S. cerevisiae*), Ensembl 51 (*H. sapiens*) and UniProt 1.0 (A. *thaliana*). The associated EC annotations were retrieved from Ensembl and UniProt. From this set we removed all proteins that were annotated with more than one EC number or with partial EC numbers. Subsequently, we discarded all EC numbers and associated proteins which were present only once in the protein collection. The final test set consists of 9,570 proteins representing 1,016 different EC numbers.

### Data

Proteome data for *Trypanosoma brucei *was obtained from the Sanger Center http://www.sanger.ac.uk. The human glutathione S-transferase was identified in the glutathion metabolic pathway in the KEGG database at http://www.genome.jp/kegg/pathway/map/map00480.html. The human protein ENSP00000196968 (Ensembl 51) was used as query for the FACT search in the yeast proteome. For the GolgA5 search, the human protein ENSP00000163416 (Ensembl 51) was used as query for the FACT search in the *T. brucei *proteome.

## Availability and requirements

**Project name**: FACT

**Project home page**: http://www.cibiv.at/FACT

**Operating system**: Platform independent

**Programming language**: Java

**Other requirements**: Java 1.5 or higher, java script enabled

## Authors' contributions

TK wrote the software and performed all analyses, IE conceived the study, TK and IE designed the study and all three authors wrote the manuscript and approved its final version.

## Supplementary Material

Additional File 1 **Supplementary Tables and Figures**. Table S1: Impact of p-value thresholds on the coverage of FACT (*FACT *score). Figure S1: Impact of p-value thresholds on the fidelity of FACT (*FACT score*). Figure S2: Cumulative fidelity along E-value thresholds for FACT (*FACT *score), BLAST and the union of FACT and BLAST. Figure S3: Venn diagram contrasting the performance of FACT (*FACT *score) and PsiBLAST. Figure S4: Contrast of PsiBLAST and FACT (*FACT *score) for different E-value/p-value combinations. Figure S5: FDP of the human GolgA5 and the highest scoring hit (MLS) in *T. brucei*: Tb927.5.1900. Figure S6: FDP of the human GolgA5 and the highest scoring hit (MS_*uni*_) in *T. brucei*: Tb11.02.4670. Figure S7: FDP of the human GolgA5 and the highest scoring hit (MS_*st*_/*FACT *score) in *T. brucei*: Tb11.02.5040. Figure S8: FDP of the human GolgA5 and the best BLAST hit in *T. brucei*: Tb11.52.0008. Click here for file
