# Supplementary material for: FACT: Functional annotation transfer between proteins with similar feature architectures
Source: BMC Bioinformatics. 2010 Aug 9;11:417. doi: 10.1186/1471-2105-11-417 (PMC2931517; doi:10.1186/1471-2105-11-417)
Supplement: Additional File 1 — Supplementary Tables and Figures. Table S1: Impact of p-value thresholds on the coverage of FACT (FACT score). Figure S1: Impact of p-value thresholds on the fidelity of FACT (FACT score). Figure S2: Cumulative fidelity along E-value thresholds for FACT (FACT score), BLAST and the union of FACT and BLAST. Figure S3: Venn diagram contrasting the performance of FACT (FACT score) and PsiBLAST. Figure S4: Contrast of PsiBLAST and FACT (FACT score) for different E-value/p-value combinations. Figure S5: FDP of the human GolgA5 and the highest scoring hit (MLS) in T. brucei: Tb927.5.1900. Figure S6: FDP of the human GolgA5 and the highest scoring hit (MSuni) in T. brucei: Tb11.02.4670. Figure S7: FDP of the human GolgA5 and the highest scoring hit (MSst/FACT score) in T. brucei: Tb11.02.5040. Figure S8: FDP of the human GolgA5 and the best BLAST hit in T. brucei: Tb11.52.0008. [file 1471-2105-11-417-S1.PDF]

Supplemental Information

p-value	max	rank 1	unique rank 1
$< 10^{-0}$	9570	8014	7091
$< 10^{-1}$	9536	8007	7084
$< 10^{-2}$	8981	7740	6849
$< 10^{-3}$	7961	6979	6185
$< 10^{-4}$	5703	5154	4574
$< 10^{-5}$	4018	3712	3290
$< 10^{-6}$	3045	2864	2513
$< 10^{-7}$	2387	2278	1992
$< 10^{-8}$	1934	1859	1620
$< 10^{-9}$	1558	1508	1317
$< 10^{-10}$	1325	1286	1114
$< 10^{-11}$	1073	1047	892
$< 10^{-12}$	909	891	760
$< 10^{-13}$	758	743	635
$< 10^{-14}$	650	636	541
$< 10^{-15}$	558	548	465

Table S1: Impact of p-value thresholds on the coverage of FACT (*FACT* score). 'max' denotes the number of searches resulting in a highest scoring protein with a p-value below the given threshold ('p-value'). 'rank 1' denotes the number of searches were the highest scoring protein has the same EC annotation as the query. 'unique rank 1' denotes the number of searches were the highest scoring protein has the same EC annotation as the query and is uniquely highest scoring.

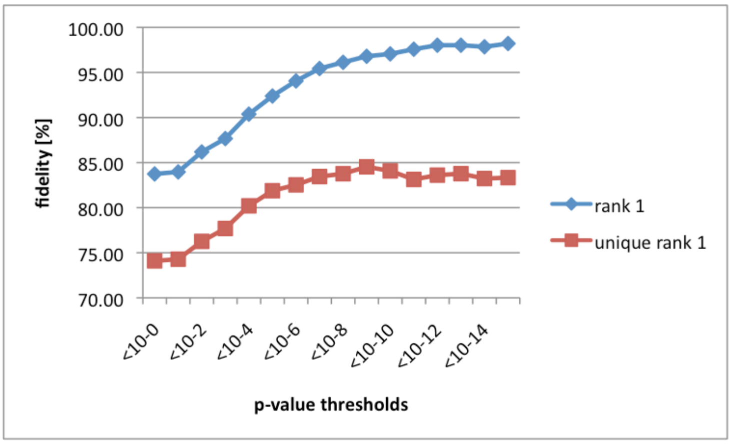

Figure S1: Impact of p-value thresholds on the fidelity of FACT (*FACT* score). 'fidelity' denotes the fraction of FACT searches where a protein with the same EC number as the query is top scoring (blue graph). The red line represents the percentage of correctly and uniquely top ranked proteins with FACT (unique rank 1). The coverage of FACT for the p-value thresholds are given in Table S1.

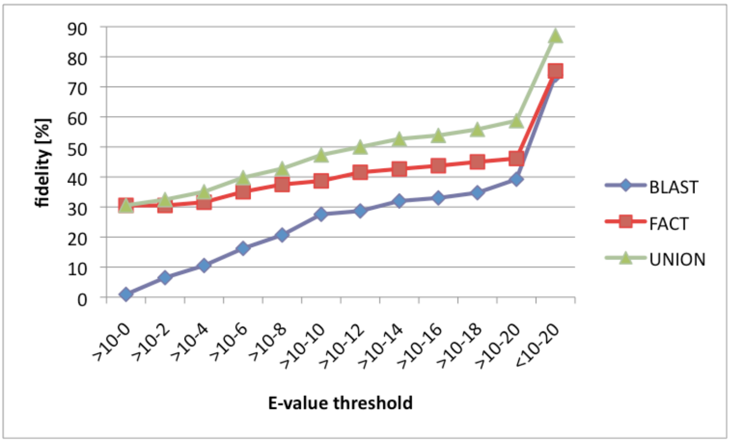

Figure S2: Cumulative fidelity along E-value thresholds for FACT (*FACT* score), BLAST and the union of FACT and BLAST.

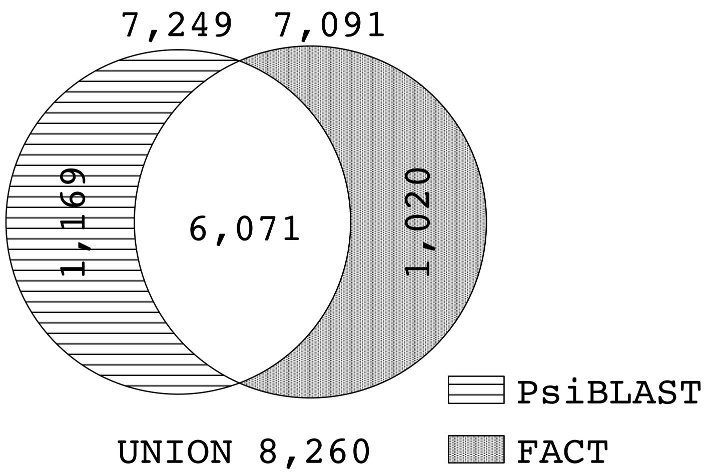

Figure S3: Venn diagram contrasting the performance of FACT (*FACT* score) and PsiBLAST. Given are the the numbers of uniquely top ranking proteins having the same EC number as the query.

p-Value \ E-value	=0	]0,10^{-15}]	]10^{-15},10^{-14}]	]10^{-14},10^{-13}]	]10^{-13},10^{-12}]	]10^{-12},10^{-11}]	]10^{-11},10^{-10}]	]10^{-10},10^{-9}]	]10^{-9},10^{-8}]	]10^{-8},10^{-7}]	]10^{-7},10^{-6}]	]10^{-6},10^{-5}]	]10^{-5},10^{-4}]	]10^{-4},10^{-3}]	]10^{-3},10^{-2}]	]10^{-2},10^{-1}]	>10^{-1}
<10-15	**462**<br>438<br>549	**3**<br>1<br>5	0<br>**1**<br>2				0<br>0<br>1									0<br>0<br>1	
]10-15,10-14]	**68**<br>62<br>82	8<br>8<br>10															
]10-14,10-13]	**93**<br>84<br>107	1<br>1<br>1															
]10-13,10-12]	**121**<br>110<br>145	4<br>5<br>6															
]10-12,10-11]	**122**<br>115<br>151	10<br>12<br>13															
]10-11,10-10]	**205**<br>189<br>227	17<br>18<br>21			0<br>0<br>1				0<br>**1**<br>1								0<br>0<br>2
]10-10,10-9]	**189**<br>167<br>209	14<br>15<br>22		0<br>**1**<br>1							0<br>**1**<br>1						
]10-9,10-8]	**276**<br>246<br>326	27<br>31<br>48						0<br>**1**<br>1									0<br>0<br>1
]10-8,10-7]	**330**<br>310<br>391	39<br>42<br>58			1<br>1<br>1	1<br>1<br>1											**1**<br>0<br>2
]10-7,10-6]	**451**<br>430<br>554	66<br>68<br>97														1<br>1<br>2	**3**<br>2<br>5
]10-6,10-5]	**630**<br>611<br>758	140<br>141<br>201	0<br>**1**<br>1				2<br>2<br>2		1<br>1<br>1							**3**<br>2<br>5	**1**<br>0<br>5
]10-5,10-4]	943<br>**951**<br>1229	**331**<br>322<br>438	1<br>1<br>1			0<br>0<br>1	0<br>0<br>1								2<br>**3**<br>3	**1**<br>0<br>2	**6**<br>1<br>10
]10-4,10-3]	1102<br>**1173**<br>1484	505<br>**517**<br>748		0<br>0<br>1											**1**<br>0<br>1		**3**<br>0<br>24
]10-3,10-2]	420<br>**485**<br>597	241<br>**274**<br>400				0<br>**1**<br>1		0<br>**2**<br>2							0<br>0<br>1	0<br>**1**<br>6	**3**<br>0<br>13
]10-2,10-1]	182<br>**258**<br>334	48<br>**98**<br>184	2<br>2<br>2	0<br>**1**<br>1		0<br>**2**<br>2	0<br>0<br>1	0<br>**1**<br>1	0<br>**1**<br>2			0<br>0<br>1		0<br>0<br>1		0<br>0<br>3	3<br>3<br>23
>10-1	0<br>**1**<br>1	7<br>**18**<br>26								0<br>**2**<br>3	0<br>0<br>1						**0**<br>1<br>2

Figure S4: Contrast of PsiBLAST and FACT (*FACT* score) for different E-value/p-value combinations. The matrix bins the 9,570 proteins according to the E-value and the p-value of the best hit when used as query for PsiBLAST and FACT, respectively. The total number of proteins for a E-value/p-value combination is given by the bottom number in the corresponding cell. The two further numbers in a cell give the number of searches FACT (top) and PsiBLAST (middle) had a functional equivalent as top scoring protein. The count number for the better performing tool is given in bold face. Yellow cells show E-value/p-value combinations where FACT identified more functional equivalents than PsiBLAST, whereas the blue cells indicate a higher fidelity of PsiBLAST. Grey cells mark ties.

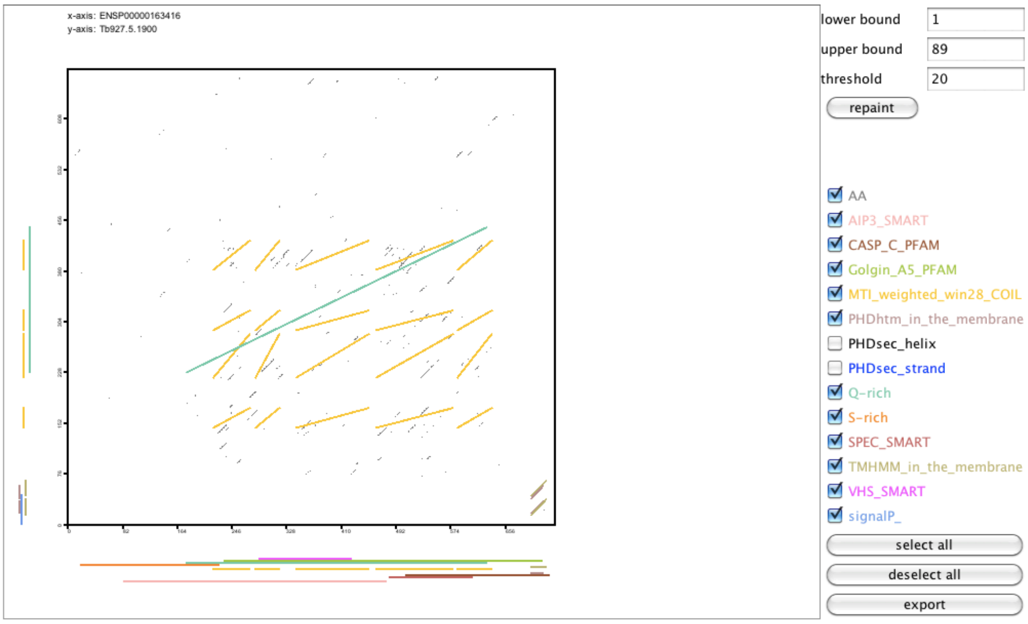

Figure S5: FDP of the human GolgA5 and the highest scoring hit (MLS) in *T. brucei*: Tb927.5.1900

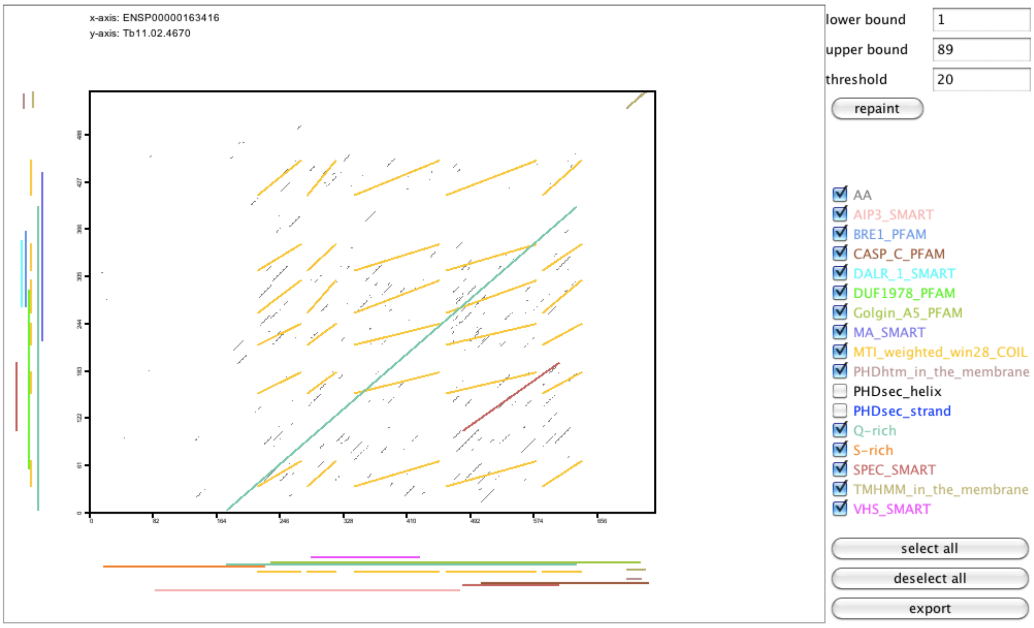

Figure S6: FDP of the human GolgA5 and the highest scoring hit (MS_{uni}) in *T. brucei*: Tb11.02.4670

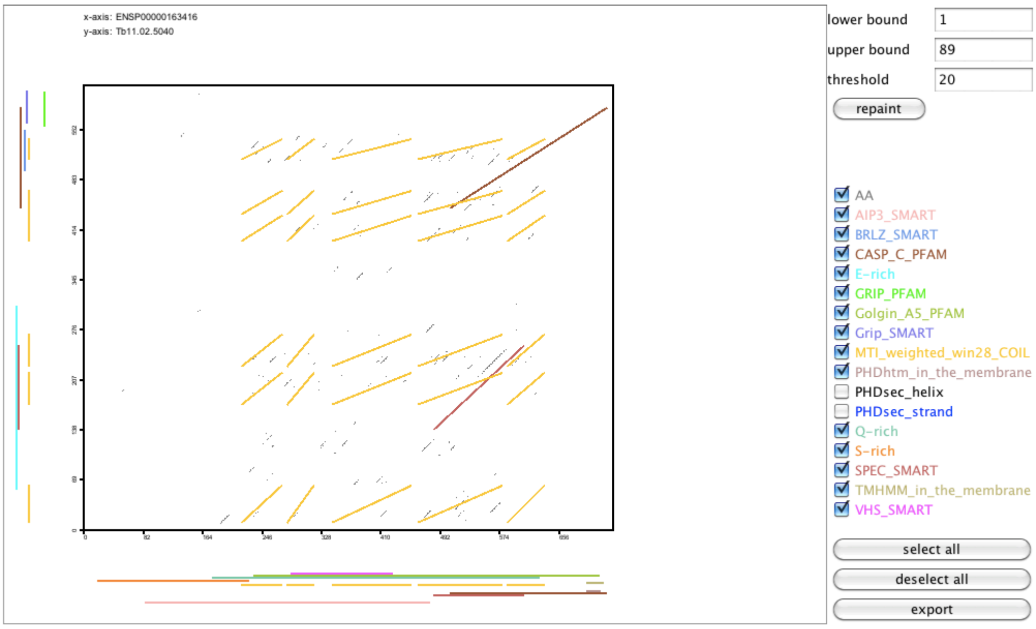

Figure S7: FDP of the human GolgA5 and the highest scoring hit (MS$_{st}$/$FACT$ score) in *T. brucei*: Tb11.02.5040

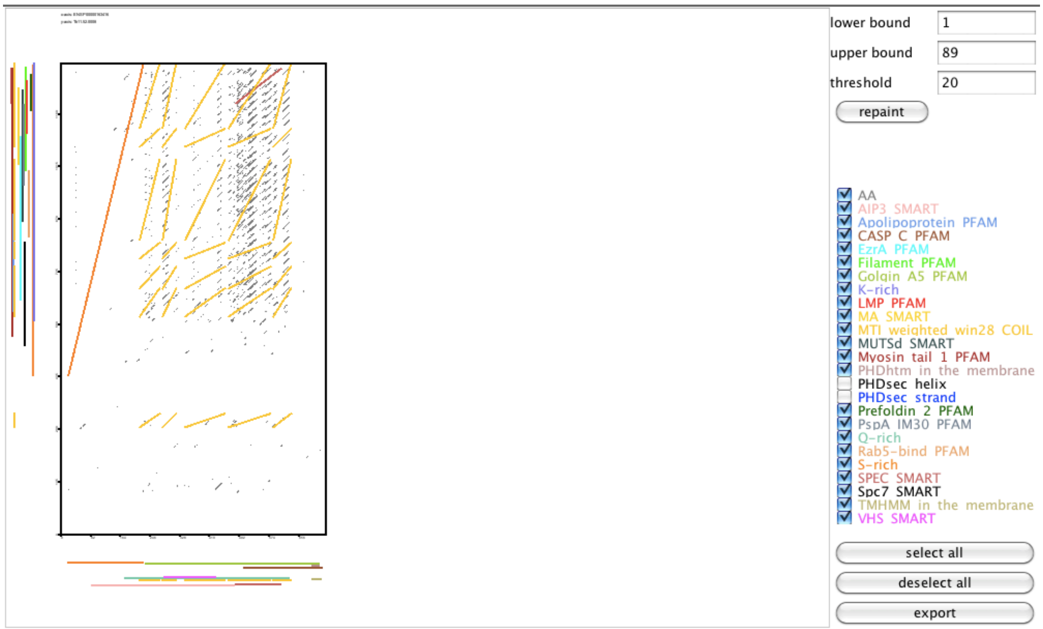

Figure S8: FDP of the human GolgA5 and the best BLAST hit in *T. brucei*: Tb11.52.0008
